# Mapping CRISPR-Cas9 public and commercial innovation using The Lens institutional toolkit

**DOI:** 10.1007/s11248-021-00237-y

**Published:** 2021-03-15

**Authors:** Osmat Azzam Jefferson, Simon Lang, Kenny Williams, Deniz Koellhofer, Aaron Ballagh, Ben Warren, Bernard Schellberg, Roshan Sharma, Richard Jefferson

**Affiliations:** 1 Cambia, GPO Box 3200, Canberra, ACT 2601 Australia; 2 Queensland University of Technology, Brisbane, Australia

**Keywords:** CRISPR, CRISPR-Cas9, CRISPR-agriculture, Agriculture, Innovation mapping, Patent, Intellectual Property, Genome editing, Gene editing, Lens.org

## Abstract

CRISPR-Cas9 is a revolutionary technology because it is precise, fast and easy to implement, cheap and components are readily accessible. This versatility means that the technology can deliver a timely end product and can be used by many stakeholders. In plant cells, the technology can be applied to knockout genes by using CRISPR–Cas nucleases that can alter coding gene regions or regulatory elements, alter precisely a genome by base editing to delete or regulate gene expression, edit precisely a genome by homology-directed repair mechanism (cellular DNA), or regulate transcriptional machinery by using dead Cas proteins to recruit regulators to the promoter region of a gene. All these applications can be for: 1) Research use (Non commercial), 2) Uses related product components for the technology itself (reagents, equipment, toolkits, vectors etc), and 3) Uses related to the development and sale of derived end products based on this technology. In this contribution, we present a prototype report that can engage the community in open, inclusive and collaborative innovation mapping. Using the open data at the Lens.org platform and other relevant sources, we tracked, analyzed, organized, and assembled contextual and bridged patent and scholarly knowledge about CRISPR-Cas9 and with the assistance of a new Lens institutional capability, The Lens Report Builder, currently in beta release, mapped the public and commercial innovation pathways of the technology. When scaled, this capability will also enable coordinated editing and curation by credentialed experts to inform policy makers, businesses and private or public investment.

## Significance statement

 The CRISPR-Cas9 system has enabled an explosion of potential new genetic engineering applications from medicine, public health, to agriculture and conservation. Concomitant with the research has been a proliferation of patents and patent applications for various uses of this technology. The toolkit described in this contribution and the insights derived from it, provide a means of navigating the complex intellectual property landscape of CRISPR-Cas9 technology.

**Hector Quemada**, *Department of Biological Sciences, Western Michigan Univ, MI, USA *

## Introduction

The idea of introducing a precise genetic change at any position within a living genome with minimal disturbance to the system is a thrilling scientific discovery. And if the technology is simple, efficient, accessible, and versatille, then its potential applications are almost limitless.

At Lens.org, we were excited to bring the contextual knowledge about CRISPR- Cas9 (scholarly, patent, human, institutional and biological), user annotations and third party content into the one place, Lens Reports, to begin the process of building maps -at various resolutions- for the innovation trajectories of this technology, mainly in the agriculture/food space and highlighting trends, blockers, or enablers across its fields of use.

Since the technological potential was articulated in the scientific literature back in 2012, there have been more than 25,000 scholarly works published on the CRISPR-Cas9 topic and over 20,000 patents filed across the globe. Enabling not only patent professionals, but also researchers, investors, policymakers, and the public to interrogate and navigate such knowledge by providing a toolkit for those trying to solve problems or help guide precise partnerships, will accelerate outcomes.

In the overview section of this report, we introduce CRISPR-Cas9 technology, which Emmanuelle Charpentier and Jennifer Doudna share the Nobel prize in Chemistry for developing, and its potential impact on the industry, intellectual property and technology transfer issues that are arising, and we offer some guidance along with some strategies for monitoring or tracking patent battles. In the scholarly analysis section, we describe the timeline of the scientific progress made in the research discipline, main scholars and their institutions driving the scientific discoveries, and compare the research outputs from China to those from the USA. This analysis is followed by a patent analysis that covers specifically the CRISPR-Cas9 system rather than the broad CRISPR technologies. We present a few patent search strategies to enable users to find information based on their interest and the issues that they are trying to address. We use dynamic Lens patent collections to highlight the most up to date information on where these filings are, who are the key applicants, and–using patent classification codes–the technology sector to which they belong. Then, we complement such analysis with a manual drawing of the licensing network across all fields of use as we were able to get information so far, and highlight the key owners of patent rights based on licensing information and press releases available online. In the last section, we perform some comparative analyses with another published broader CRISPR patent landscape and show various trends based on various technology sectors. All the data presented in the report are then made available in the Data sources section and can be linked to, cloned, and shared with others. The draft report is a prototype of Lens.org that is being developed at present, and we are providing examples for the larger community to seek their feedback on the usefulness of such a toolbox in their own workplace and to engage them in building a more transparent innovation ecosystem.

## Overview

### Why is CRISPR-Cas9 a revolutionary technology?

CRISPR-Cas9 is a revolutionary technology because it is precise, fast and easy to implement, cheap, and uses components readily accessible. This versatility means that the technology can deliver a timely end product and can be used by many stakeholders.

In plant cells, the technology can be applied to knock out genes by using CRISPR–Cas nucleases that can alter coding gene regions or regulatory elements, alter precisely a genome by base editing to delete or regulate gene expression, edit precisely a genome by homology-directed repair mechanisms (cellular DNA), or regulate transcriptional machinery by using dead Cas proteins to recruit regulators to the promoter region of a gene (https://www.nature.com/articles/s41477-019-0461-5). All these applications can be for:

1. Research use (Non commercial)
2. Uses related product components for the technology itself (reagents, equipment, toolkits, vectors etc.)
3. Uses related to the development and sale of derived end products based on this technology

### Intellectual property and technology transfer issues

Public disclosure of patented inventions and diverse technology transfer models are critical to advance the application of these technologies in society. Sherkow and Scott (2019) highlighted some of the issues that relate to the various components of the broad genome editing technologies, including CRISPR, wherein secrecy is used to hide some parts of the technology and patenting other elements to tie up and create a dependency on a product which can then maximize profits. And recently, Graff and Sherkow (2020) reviewed and analyzed university-based technology transfer models and found that diversity may be advantageous as it may create several substitutes to some of the claimed inventions, allowing a broader use and commercialization. Currently, there are over 100 licenses or sub licenses discovered at the genomeweb.com site. These are publicly disclosed, but many others are not, according to personal communication with industry colleagues. Below we show a map of the early 60 licenses identified in this study.

**Fig. 1:**
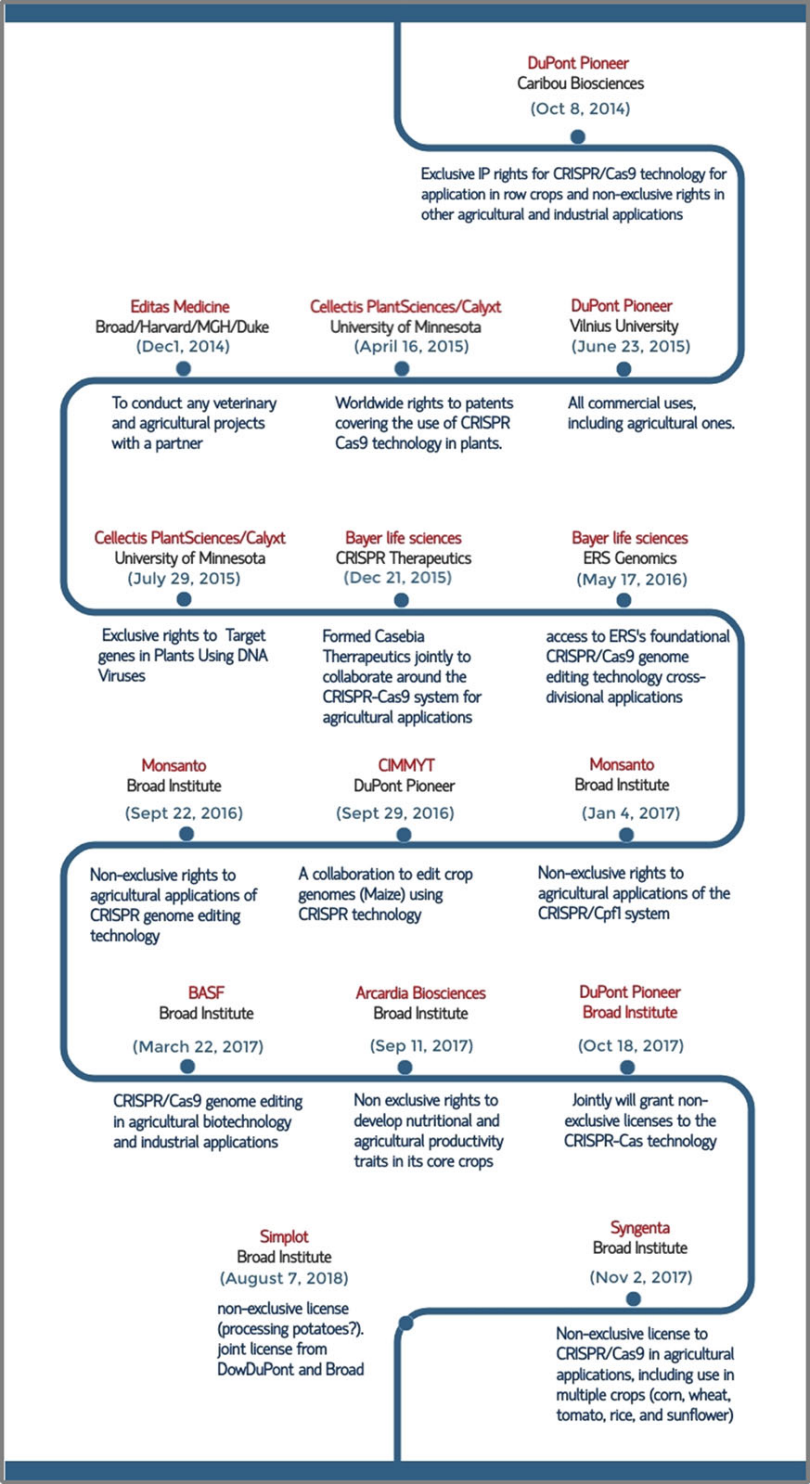
Timeline for the early CRISPR licenses-Agriculture*. *Data source: dataverse.harvard.edu and genomeweb.com. Accessed on November 8, 2020

Giant agriculture companies such as DuPont Pioneer, Monsanto (merged with Bayer), BASF and Syngenta seem to be the early licensees and in most cases, they licensed different CRISPR technologies from the various IP holders, gaining access to the latest research tools available (Fig. 1). The category of applications identified so far includes: Human Health (20) Animals (3) Agriculture (15), and Technical improvement (22) Licensing agreements varied from research collaborations, partnerships, simple commercial use, to joint ventures, acquisitions and mergers.

### Tracking CRISPR patent battles

**Fig. 2:**
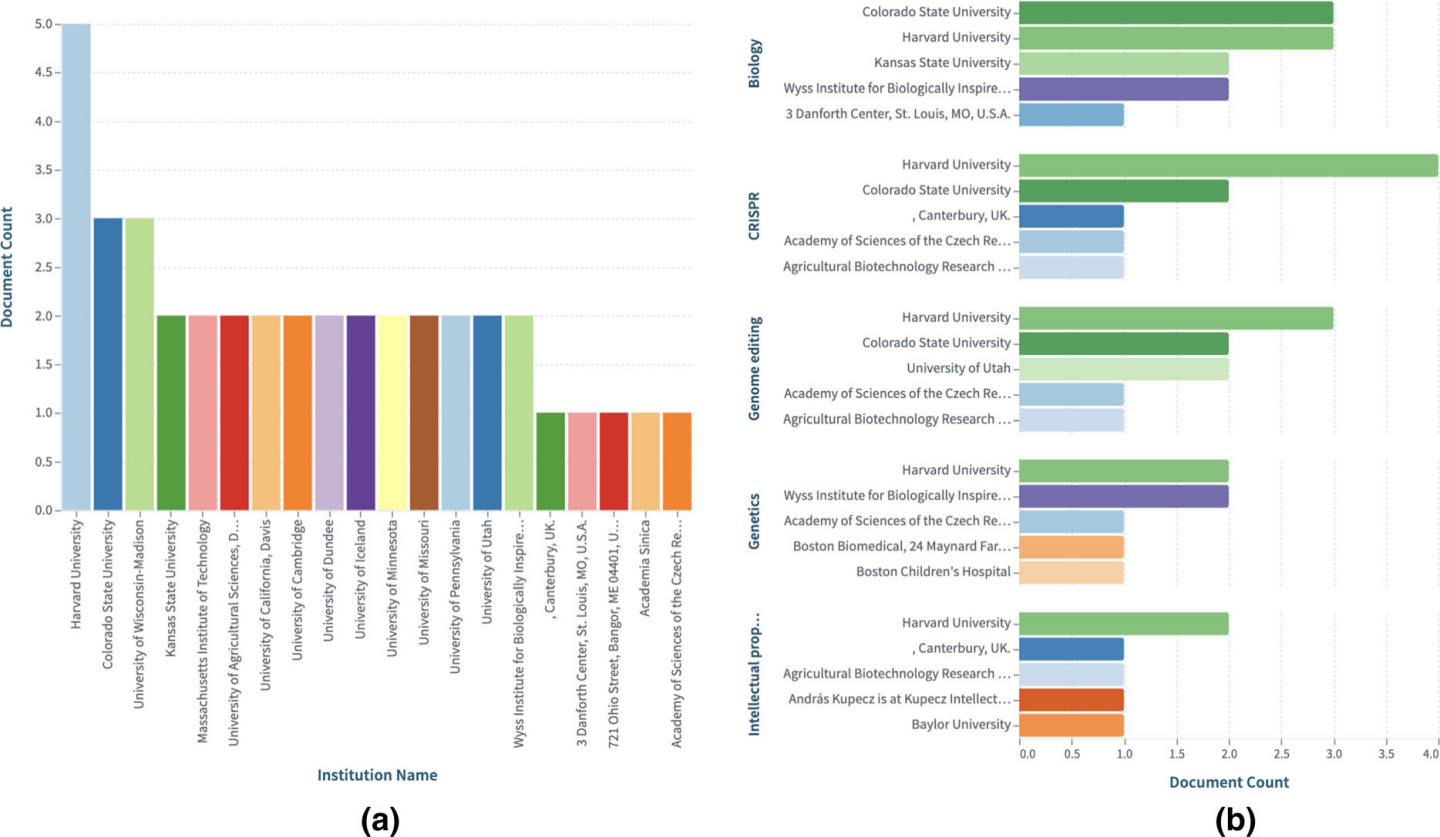
Top affiliated Institutions with scholarly works addressing Intellectual property and technology transfer issues regarding the CRISPR-Cas9 technology (**a**), and when the scholarly works collection was first filtered by Field of Study, we show the groupings of these affiliated institution by that field (**b**). Readers can explore further the dynamic collection in the Lens at https://link.lens.org/YoTUeNdu3oh and follow the dynamic mapping in the online report at https://link.lens.org/Wca170NGgqg

The long-running patent battle between the CRISPR team at the University of California (UC) and that at the Broad institute over who was the first to invent uses of the original CRISPR system in eukaryotic cells continues to rage. While in Europe the UC team leads, in the USA, the Broad institute leads. The current status of the intellectual property rights decisions can be searched by entering this string in the Lens: author.display_name:("Jon Cohen") AND (CRISPR). Automatic updates can be set up by saving the string in your work area and requesting alert notifications. Scholarly works addressing intellectual property and technology transfer challenges and opportunities of CRISPR-Cas9 system, can be found in this dynamic collection, CRISPR Cas9: IP & Tech transfer that is based on this search query: (title:("CRISPR Cas9") OR abstract:("CRISPR Cas9") OR fulltext:("CRISPR Cas9")) AND ((title:("Intellectual property") OR abstract: ("Intellectual property") OR fulltext:("Intellectual property")) OR (title:("IP landscape") OR abstract:("IP landscape") OR fulltext:("IP landscape"))) and which has been manually edited to select what we thought are the most relevant works (Fig. 2). Others are welcome to edit the search, modify it and share it. In this report, we analyze various sets of patent searches related to the technology, and compare findings from a recent landscaping exercise, with results in the Lens enabling the user to access these datasets from Lens.org for further analyses.

## CRISPR Cas9 Scholarly works analysis

### Who is investing in CRISPR Cas9 research ?

**Fig. 3:**
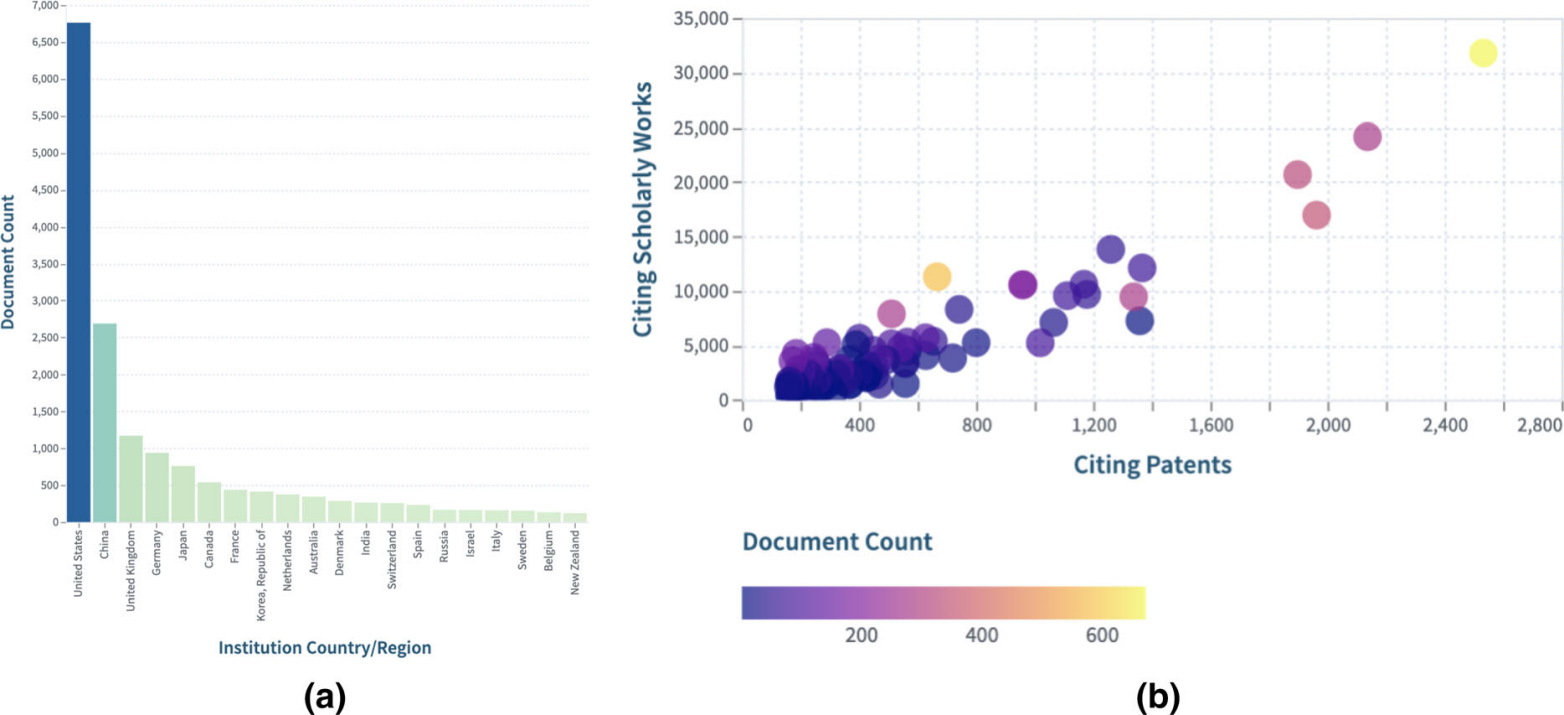
**a** Scholarly works contributions by country and **b** Citation comparison per institution.

Figure 3a shows countries that are contributing scientific knowledge on this research tool and Fig. 3b displays the extent of the scholarly and patent citations on such scientific knowledge. Readers can view the dynamic collection in the Lens at https://link.lens.org/mnbBUVIsulg.

Identifying which countries are contributing to CRISPR-Cas9 technology allows different actors in the innovation system to find each other, to better understand the capabilities each brings, to find and share opportunities to collaborate and formulate more productive partnerships, to minimize duplicated efforts, unnecessary intellectual property risks, and guide ongoing decisions on forward trajectories. To create a search strategy, an initial scouting was done of various keywords and concepts around that technology and after a few preliminary searches, this simple broad search was developed: title:CRISPR-Cas9 OR abstract:CRISPR-Cas9 OR keyword:CRISPR-Cas9 OR field_of_study:CRISPR-Cas9 OR title:cas9 OR abstract:cas9 OR keyword:cas9 OR field_of_study:cas9 OR (title:(CRISPR) OR abstract:(CRISPR) OR keyword:(CRISPR) OR field_of_study:(CRISPR)). This resulted in a dynamic parent collection: CRISPR Cas9: Broad. To narrow it further to the CRISPR Cas9 system, The search results were filtered using the "Field of Study" facet: CRISPR and Cas9, and a sub collection named: CRISPR Cas9: Field Of Study CRISPR + Cas9 was developed, which was then used to do the preliminary analyses.

### Which scientific works are perceived most important?

Importance of scholarly works here is tracked based on the cited scholarly works in the patent and scholarly literature. We list below the top three publications based on the number of their unique citing patents or citing scholarly works using CRISPR Cas9: Field Of Study CRISPR + Cas9 collection.

#### Top scholarly works based on either unique citing patents or scholarly work counts:

- A programmable dual-RNA-guided DNA endonuclease in adaptive bacterial immunity. Martin Jinek, Krzysztof Chylinski, Ines Fonfara, Michael Hauer, Jennifer A. Doudna, Emmanuelle Charpentier. Science, Volume: 337, Pages 816–821. August 17, 2012. 1,405 Citing Patents, 6,894 Citing scholarly works,
- Multiplex Genome Engineering Using CRISPR/Cas Systems
  Le Cong*,* F Ann Ran*,* David D Cox*,* Shuailiang Lin*,* Robert P J, Barretto*,* Naomi Habib*,* Patrick D Hsu*,* Xuebing Wu*,* Wenyan Jiang*,* Luciano A Marraffini*,* Feng Zhang. Science, Issue: 6121, Volume: 339, Pages: 819–823. Jan 3, 2013. 1,056 Citing Patents, 8,061 Citing Scholarly Works,
- RNA-Guided Human Genome Engineering via Cas9. Prashant Mali*,* Luhan Yang*,* Kevin M Esvelt*,* John Aach*,* Marc Guell*,* James E DiCarlo*,* Julie E Norville*,* George M Church. Science, Issue: 6121, Volume: 339, Pages: 823–826. Jan 3, 2013. 886 Citing Patents, 5,471 Citing Scholarly Works,

### Which institutions are most active and since when?

**Fig. 4:**
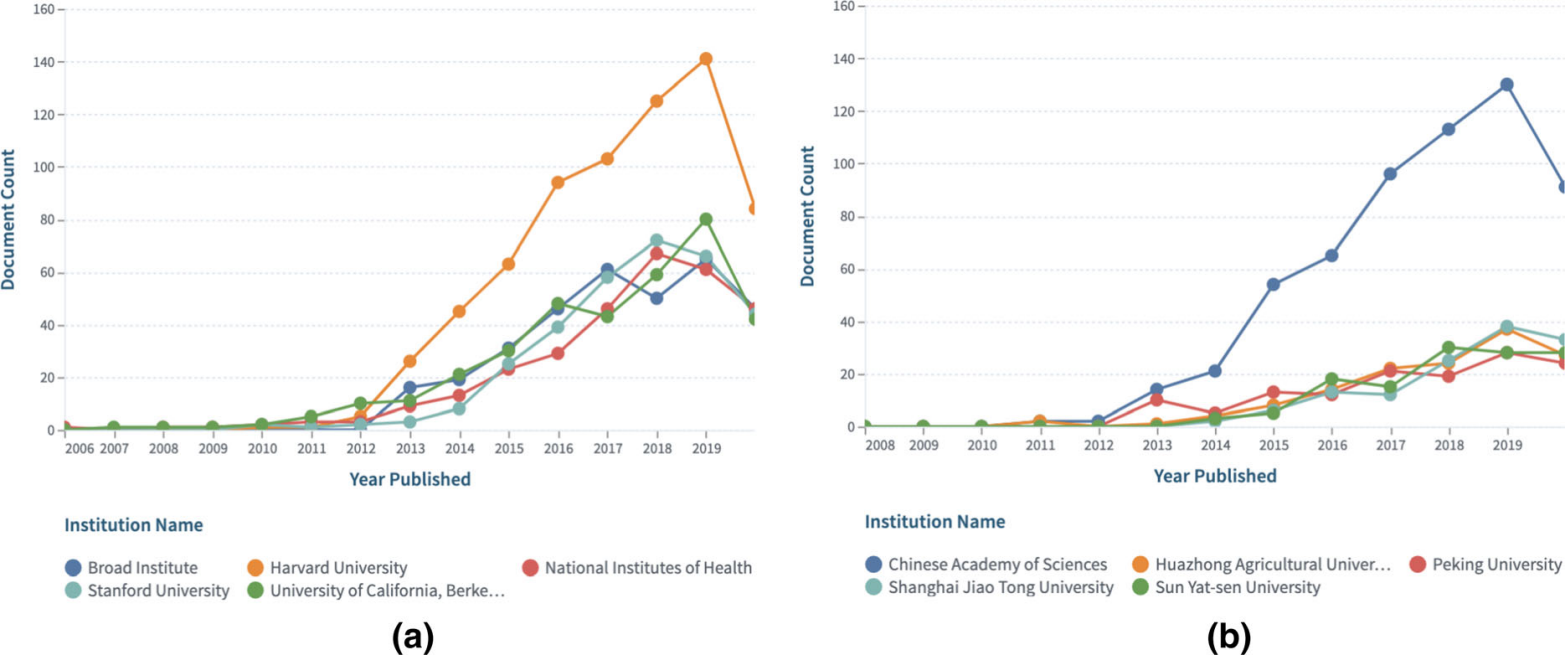
**a** Top US institutions research over time (https://link.lens.org/9z8bAJd7l8k) and **b** Top Chinese institutions research over time (https://link.lens.org/PPYHGQDN8i)

**Fig. 5:**
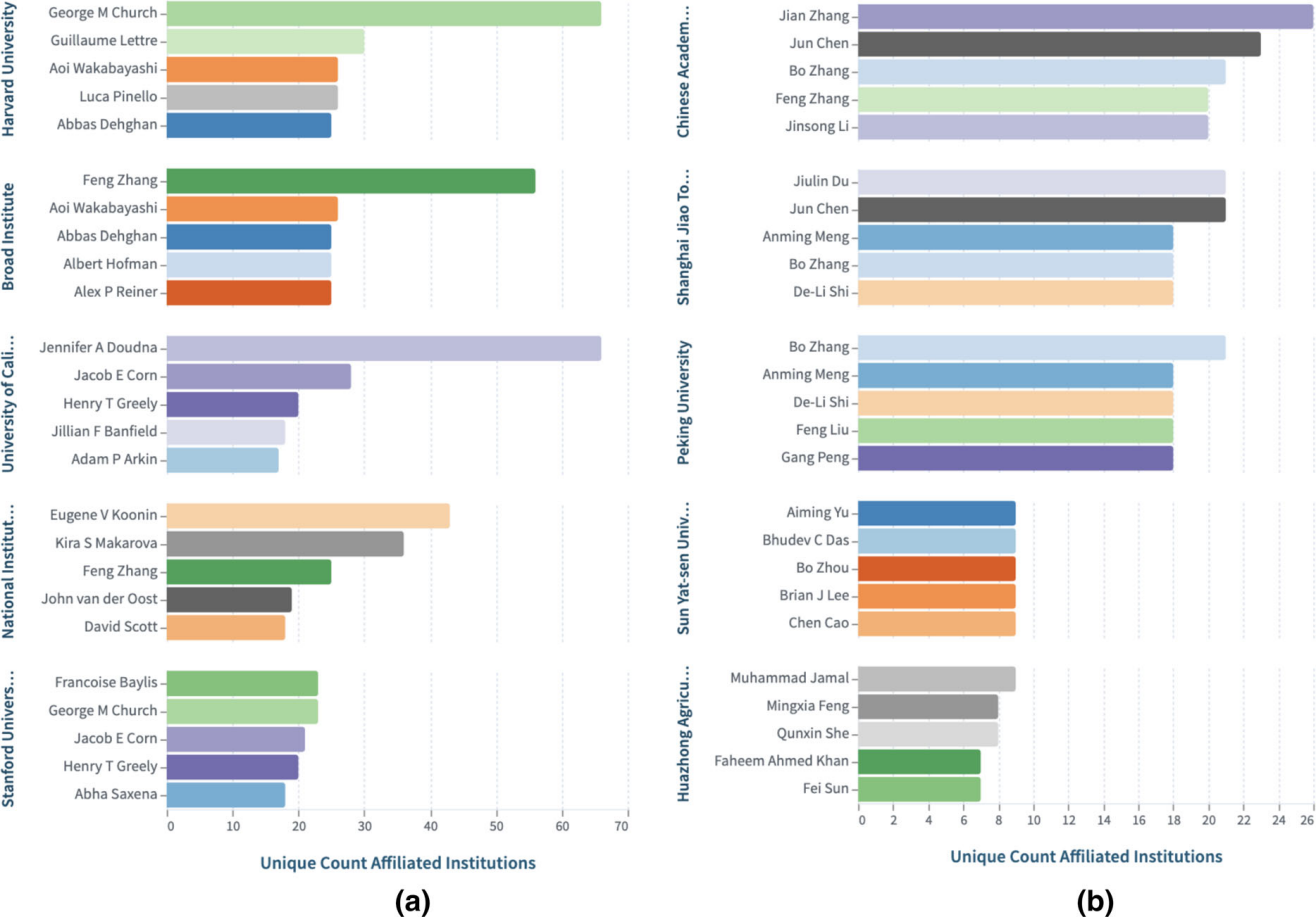
**a** Most active authors per US institution, and **b** Most active authors per Chinese institution. Readers can explore further the US and Chinese collections in the Lens.

The collection CRISPR Cas9: Field Of Study CRISPR + Cas9 was filtered further by either USA to generate the CRISPR Cas9: USA collection or China to generate the CRISPR Cas9: China collection and both collections were then compared and analyzed over time and space (Figs. 4 and 5). Research related to the CRISPR-Cas9 fields of Study seems to have started as early as 2006 in the USA compared to 2010 in China.

### Who is doing the research at these institutions?

The most active authors are displayed based on the institutional affiliation recorded in the scholarly work results set and may include collaborating institutions (Fig. 5).

## Analysis of patents related to CRISPR-Cas9 technology field

This analysis targets the CRISPR-Cas9 system, rather than the broad CRISPR-based gene editing. For a comparative analysis of the latter please see the next section.

To build on the early patent landscape by Egelie et al., 2016 and capture inventions that claim the CRISPR-Cas9 system including any of its components, we used this simple search strategy that highlights relevant keywords in the patent claims: claims:CRISPR OR claims:Cas9 OR claims:gRNAs OR claims:("RNAs guided") OR claims:("guided RNAs") OR claims:sgRNAs OR claims:("CRISPR cas9"). Search results captured 7,980 Patents (3,835 Families) on October 9, 2020 which were then used to create the dynamic collection, CRISPR Cas9: Claims. This collection was then expanded by simple patent family (CRISPR Cas9: Claims-Expanded) to capture all filings around the world and determine geographical distribution of potential patent rights, should patent applications be issued.

An alternative/complementary search strategy was also tried by narrowing the search to just the keyword "CRISPR Cas9" in the claims, or by combining it with a search for a CPC classification code C12N2310/20* taking into consideration the use of examiner-classified code for CRISPRs which may reveal new patents. The resulting search string used in the Lens: claims:(CRISPR cas9) OR classification_cpc: (C12N2310/20*).

Both search results were saved into dynamic collections: (1) CRISPR Cas9: only in claims, and (2) CRISPR Cas9: claims & CPC code. Both results show that the majority of documents are patent applications and most filings have been recent, indicating that the technology is still in its early stage of development. For a more detailed mapping and landscaping, readers may want to try various search strategies in the Lens, edit or combine various queries and using the various Lens tools, distill these into a set of patents which can then be saved into a dynamic collection for further analysis/ tracking, and manual inspection.

### Who is inventing and participating in this technology?

**Fig. 6:**
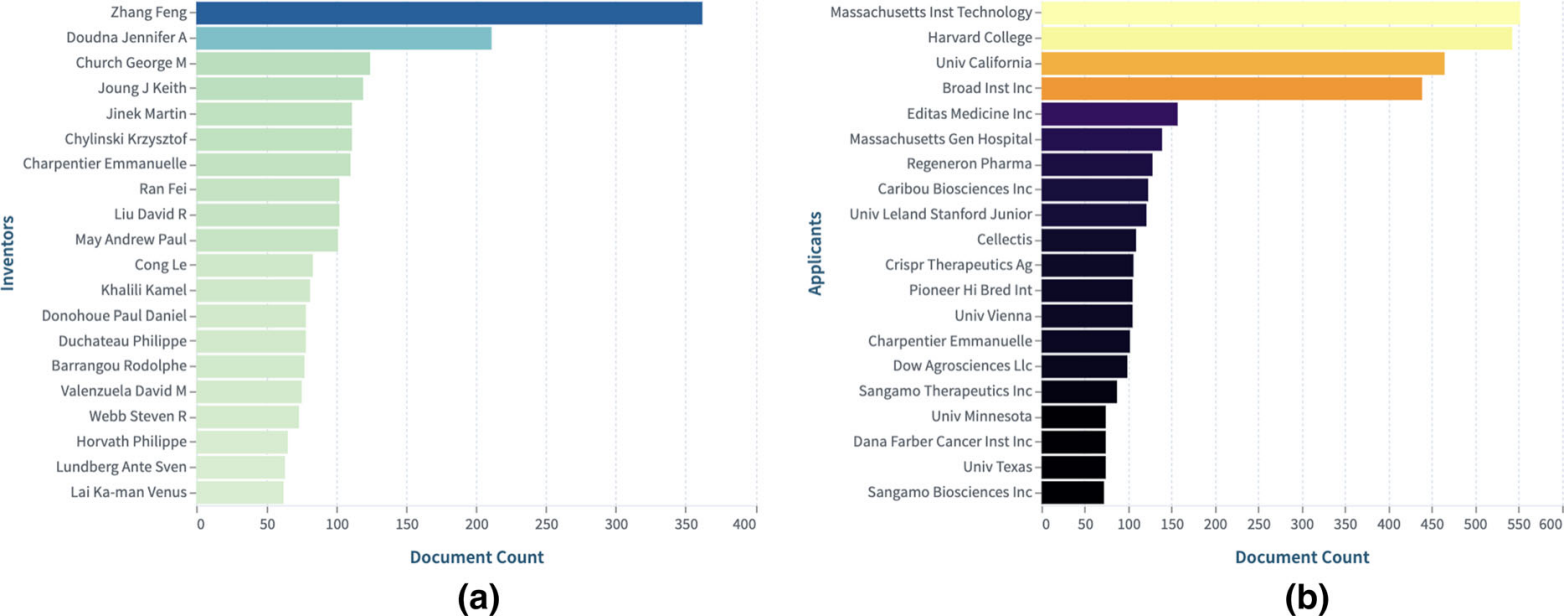
**a** Top inventors, and **b** Top patent applicants in the CRISPR-Cas9 field.

Universities seem to be leading the patenting race but are they also participating in product development? Fig. 6 displays the top inventors and applicants based on Lens dynamic collection: CRISPR Cas9: Claims (https://link.lens.org/LLl1Aea0RVb).

#### Where are these patents filed?

**Fig. 7:**
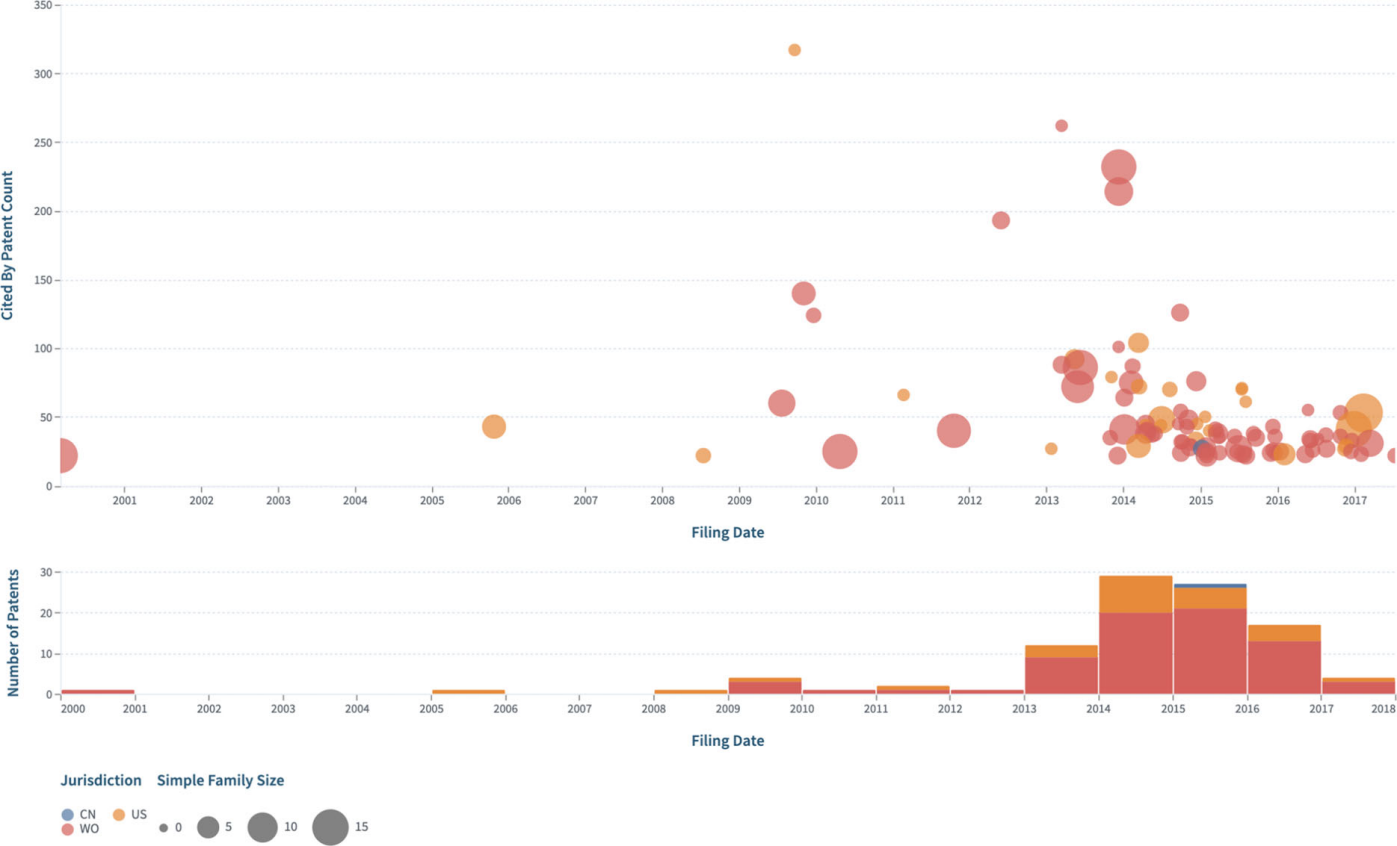
Patent scatter plot sorted by forward patent citations and based on filing date

**Fig. 8:**
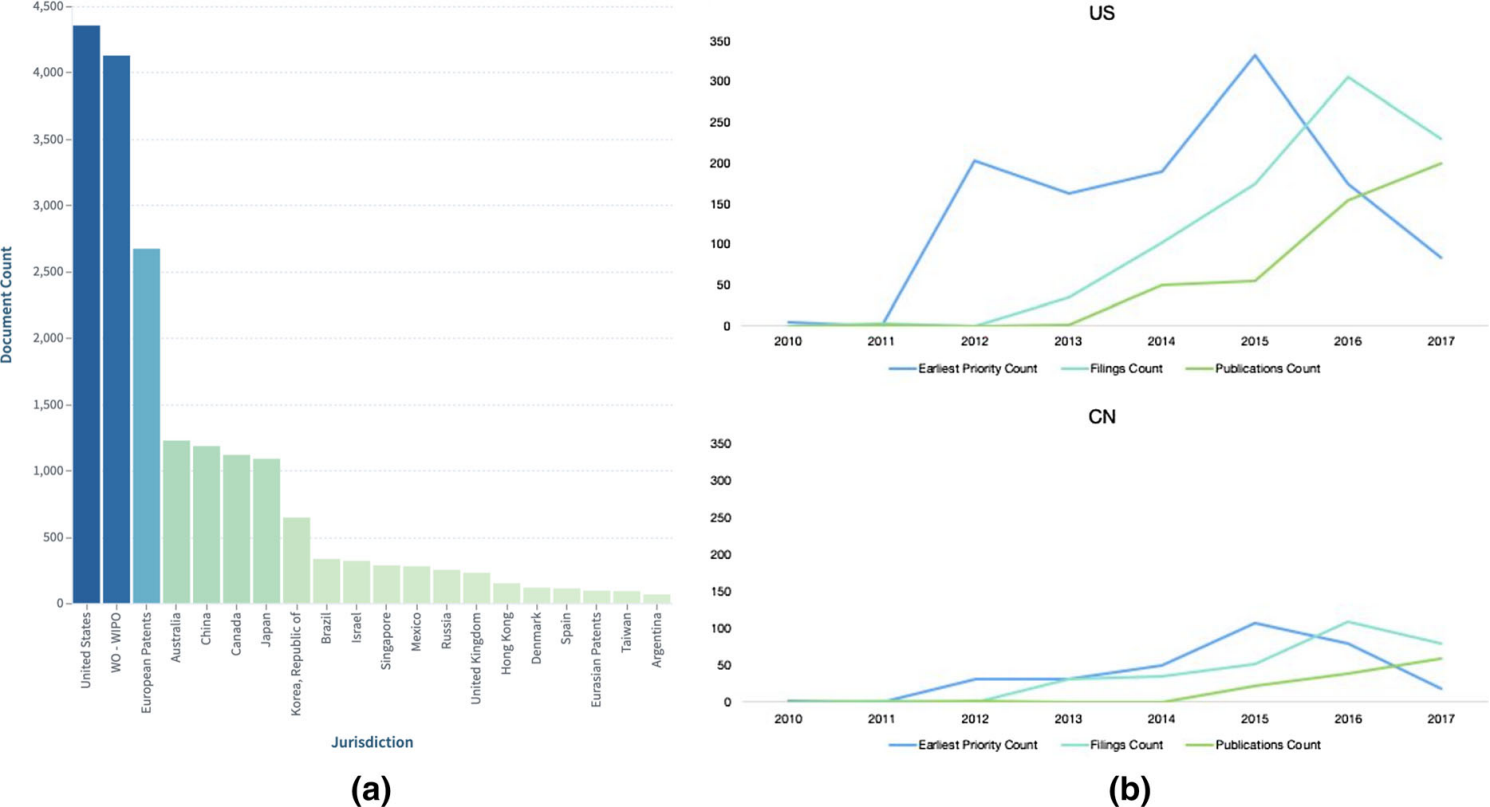
Displays **a** Top jurisdictions where patents were published and **b** trends of document counts by publication, filing, and earliest priority year in US versus China

**Fig. 9:**
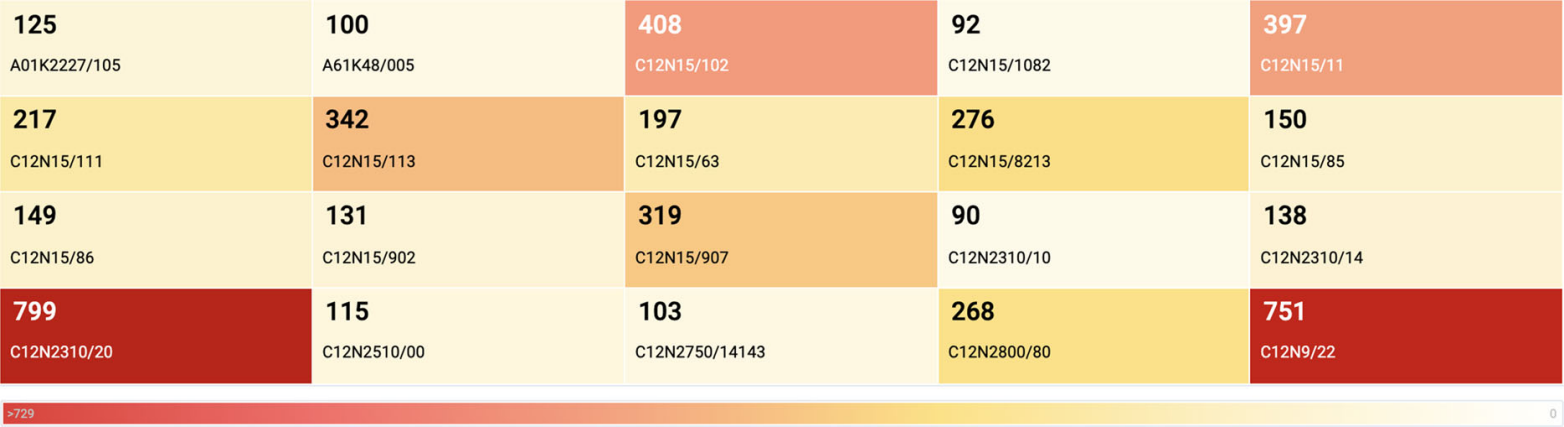
Top 20 fields of Technology

Analysis of the Lens dynamic collection, CRISPR Cas9: Claims expanded by patent family, showed that global filings accelerated post-2013. The majority of the top 100 patents were international filings (WO) and were cited by other patents. Forward patent citations ranged from 1 to more than 300 (Fig. 7). Further analysis reveals that international filings are almost on par with US publications. Readers can explore further the graphs from Figs. 7, 8 and 9 and the corresponding collection in the Lens and create their own customized dashboard.

#### What are the main technology fields of use covered ?

Here, we display the technology fields of use based on The Cooperative Patent Classification (CPC) which is a joint effort between the United States Patent and Trademark Office (USPTO) and the European Patent Office (EPO) to harmonize their existing classification systems (Fig. 9). These codes are generally assigned by patent examiners to reveal the nature and field of use of the claimed invention in a patent and readers can look up the verbose meaning of the codes in the Lens Classification Explorer when viewing this graph in the Lens.

#### Which patents are the most important?

Importance can be measured by the size of the simple patent family, a value, mainly perceived by patent applicants upon increasing the filings of their patent applications, considered to be the same inventions, across various jurisdictions. Also, importance can be evaluated by whether the patent was recognized and cited by other patents, i.e.the number of forward patent citations. As an example, here are the top two patents based on each of the two metrics.

1. Top valued patents based on family size
  - Methods And Compositions For Rna-directed Target Dna Modification And For Rna-directed Modulation Of Transcription. Published: Jun 29, 2017 Filed: Mar 15, 2013. Earliest Priority: May 25, 2012. Applicant: CHARPENTIER EMMANUELLE, UNIV CALIFORNIA, UNIV VIENNA. **Family: 204** Cited Works: 1 Cited by: 0 Cites: 2. Granted Patent: AU 2013/266,968 B2, Lens ID: 044–218-111–367-891
  - Crispr-based Genome Modification And Regulation. Published: Jun 4, 2015 Filed: Dec 5, 2013 Earliest Priority: Dec 6, 2012 Applicant: SIGMA ALDRICH CO LLC. **Family: 105** Cited Works: 0 Cited by: 0 Cites: 0. Patent Application: AU 2013/355,214 A1, Lens ID: 069–897-985–352-951
2. Top valued patents by whether they are cited by other patents
  - Target Dna Interference With Crrna. Published: Mar 25, 2010 Filed: Sep 23, 2009 Earliest Priority: Sep 23, 2008. Applicant: UNIV NORTHWESTERN. Family: 1 Cited Works: 0 **Cited by: 317** Cites: 1. Patent Application: US 2010/0,076,057 A1, Lens ID: 163–191-723–263-652
  - Rna-directed Dna Cleavage By The Cas9-crrna Complex. Published: Sep 26, 2013 Filed: Mar 15, 2013. Earliest Priority: Mar 20, 2012. Applicant: UNIV VILNIUS. Family: 30 Cited Works: 39 **Cited by: 262** Cites: 3. Patent Application: WO 2013/141,680 A1, Lens ID: 030–123-260–799-09X

#### How does the licensing network across all fields of use look like?

The CRISPR licensing network can be explored at.

https://public.flourish.studio/visualisation/1011791/embed. By clicking on any of the blue circles, one can view the licensor and licensees in this interactive network. Data Source: CRISPR Licenses Dataverse. Network graph by Flourish team.

##### Who has IP rights to Agricultural applications using CRISPR technology?

**Fig. 10:**
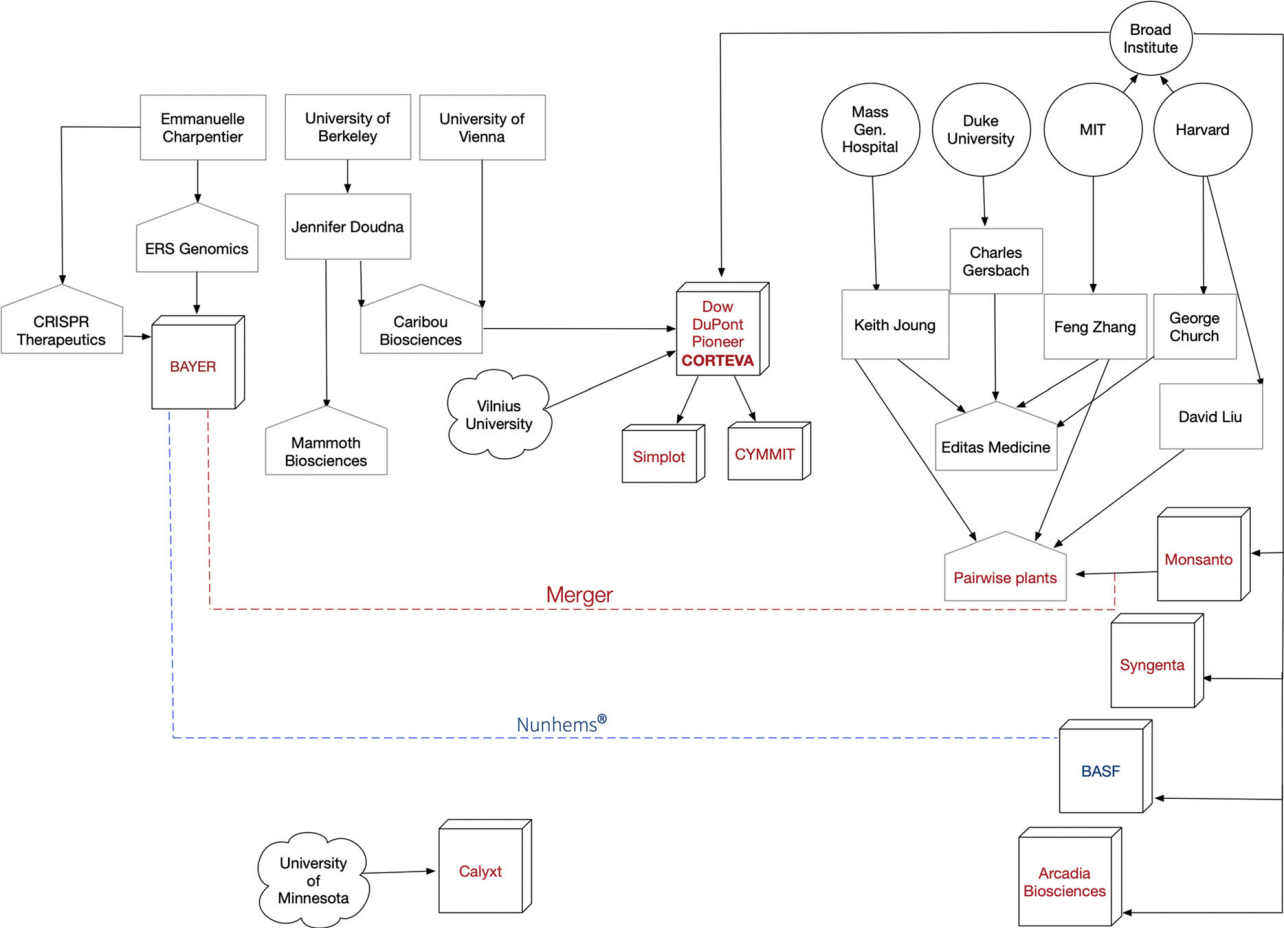
CRISPR-IP holders-Surrogate companies-Agriculture licensees. Red dotted lines represent the current merger between Bayer and Monsanto and the blue dotted line represents the relationship between Bayer and BASF. Such relationships may have broad implications for access to CRISPR-Cas9 technologies from both the UC group and the Broad Institute and among themselves. Similarly, one can see how Corteva has managed to secure licenses from both leading players with the rights to sub-license to even international research organizations and other companies

Exclusive and non-exclusive worldwide licenses have been granted to the major agriculture companies and structural re-organizations of some of these companies have been observed since 2017. For example, BASF signed agreements with Bayer in 2017 and 2018 to buy its global vegetable seeds business, mainly operating under the brand Nunhems®. Bayer and Monsanto have merged and although integration is taking place, the two companies will be operating as two separate legal entities in many countries for several years. Moreover, the spinoff of DuPont Pioneer, DuPont Crop Protection and Dow AgroSciences, Corteva Agriscience™ has become independent and publicly traded company since June 1, 2019 (Fig. 10).

## What trends other published CRISPR patent landscapes show?

**Fig. 11:**
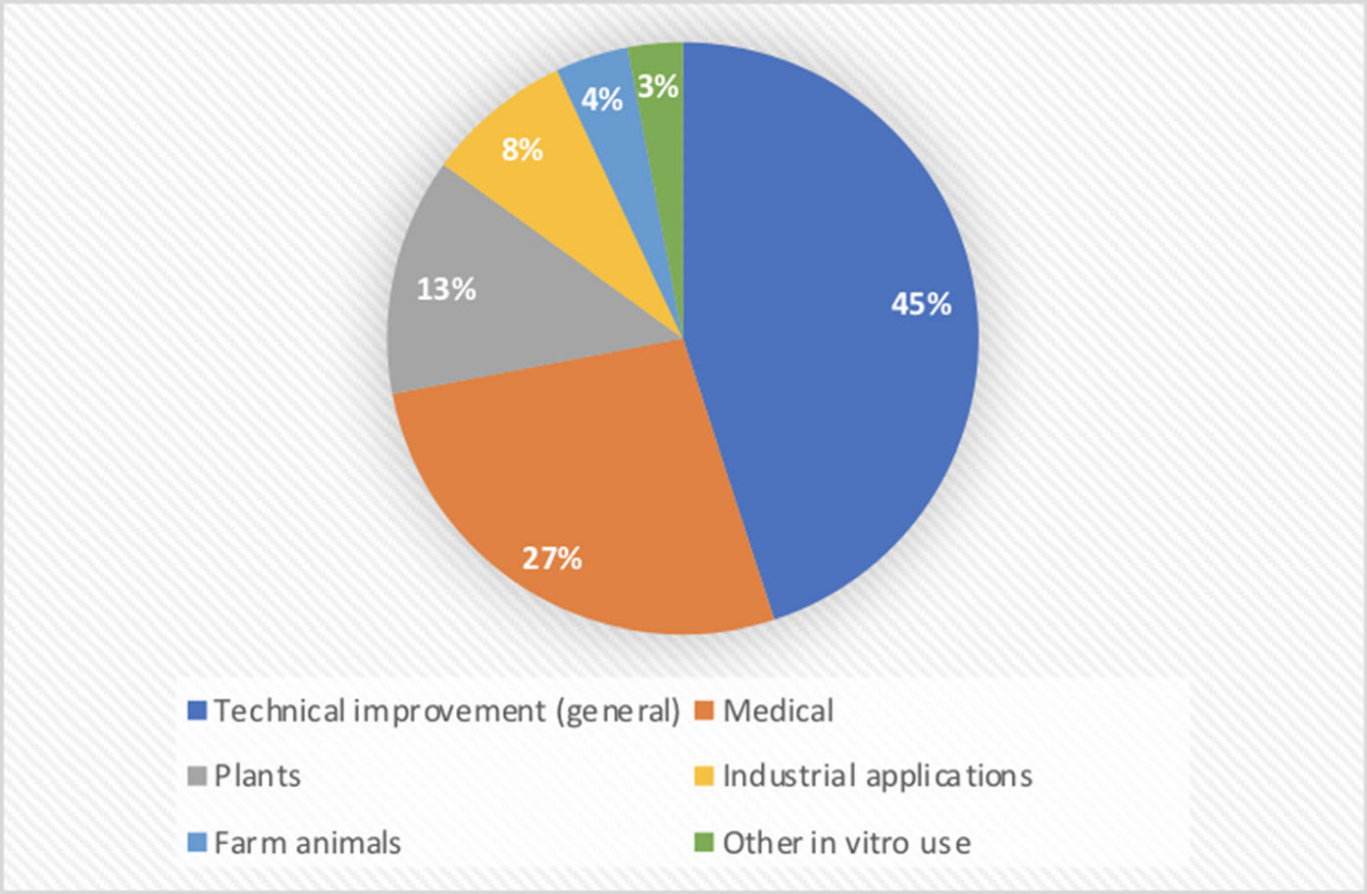
Technical categories within the CRISPR system patent collections as published in Martin-Laffon et al. 2019

Martin-Laffon et al.,2019 reported a manually curated "per technical categories'' related inventions list (Fig. 11), with a total of 2072 patent families considered as **CRISPR gene editing patents** (from 2002 up to 31 December 2017 as a priority date). We extracted the published patent publication keys from that list and created an overall Lens patent collection that encompasses all categories and separate collections for each technical category (Table 1). As the published data was based on early patent filings (patents with the earliest priority data number), we used either the extracted documents list for each technical category to show geographical distribution and applicants based on the earliest filed patent documents or the expanded by simple patent family documents to capture all subsequent filings that are published. The analyses show a slight shift in the most active jurisdictions and applicants in some of the categories over time suggesting increased patenting activities across various jurisdictions following early filings (Figs. 12, 13, and 14). Similarly, applicants' proportions also shifted with time. To view the criteria the authors used to select patents please see the supplementary note in their publication at https://static-content.springer.com/esm/art%3A10.1038%2Fs41587-019-0138-7/MediaObjects/41587_2019_138_MOESM1_ESM.pdf

**Table 1:** Published patent families as reported by Martin-Laffon et al. (2019) and extracted by the Lens team

Technical Category	Published patent families*	Lens reconstructed families	Lens Public collection (families)
Technical improvements	942	940	CRISPR:Technical improvement
Medical	554	563	CRISPR-Medical
Plants	267	261	CRISPR: Plants
Animals + Aquaculture	85	86	CRISPR: Animals
Industrial applications	167	168	CRISPR: Industrial applications
Others	57	52	NA
All categories	2072	2070	CRISPR: Technical categories

*Martin-Laffon et al. (2019)

### Trends across all technical categories (https://link.lens.org/8puu7APdjD) See Fig. 12

**Fig. 12:**
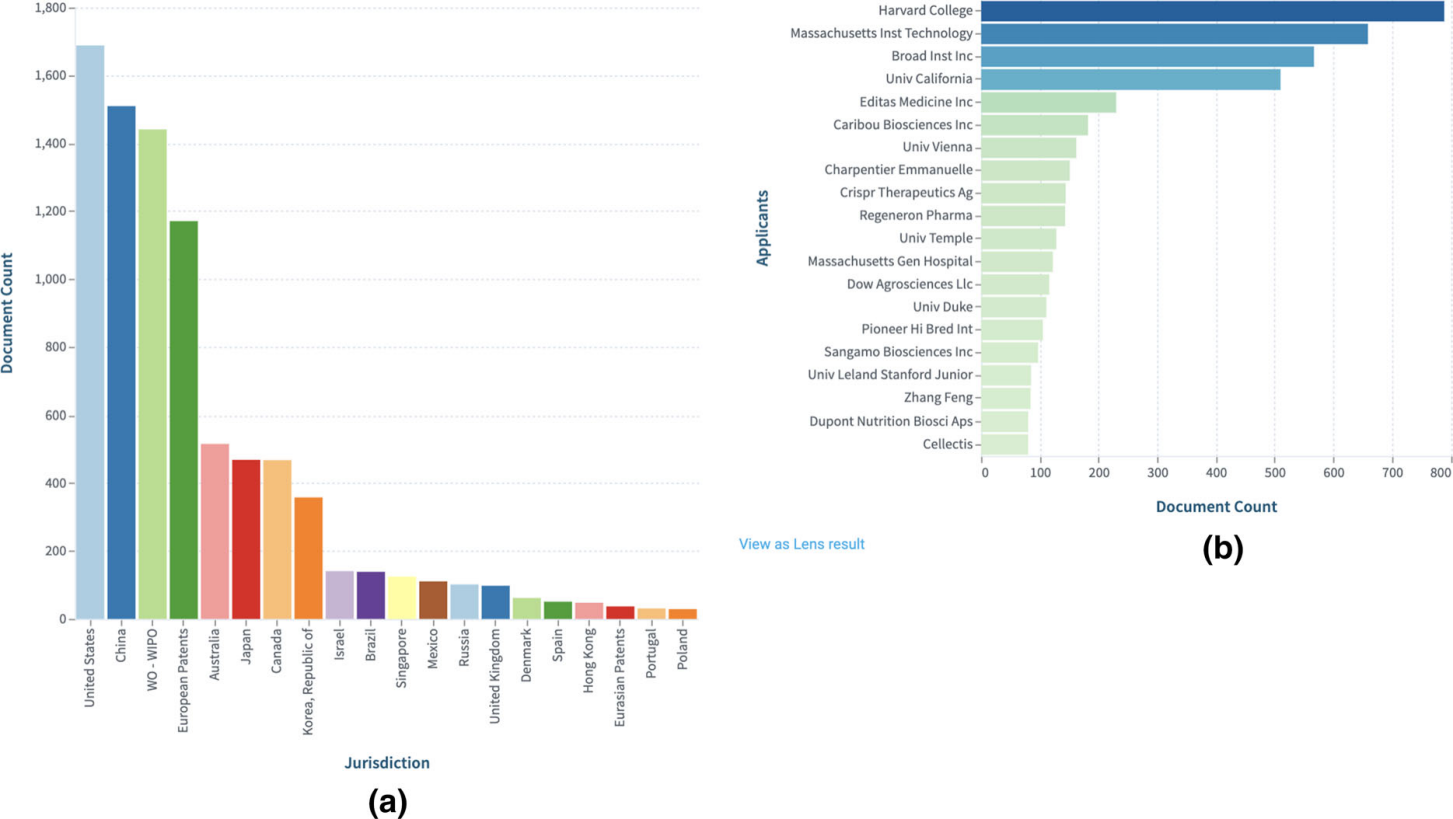
**a** Top jurisdictions based on expanded patent families, and **b** Top applicants based on expanded patent families. Explore further CRISPR: All technical categories-Expanded by simple patent family in the Lens.

### Trends in "Agriculture/Plants" category (https://link.lens.org/so6kkI4SBPc) See Fig. 13

**Fig. 13:**
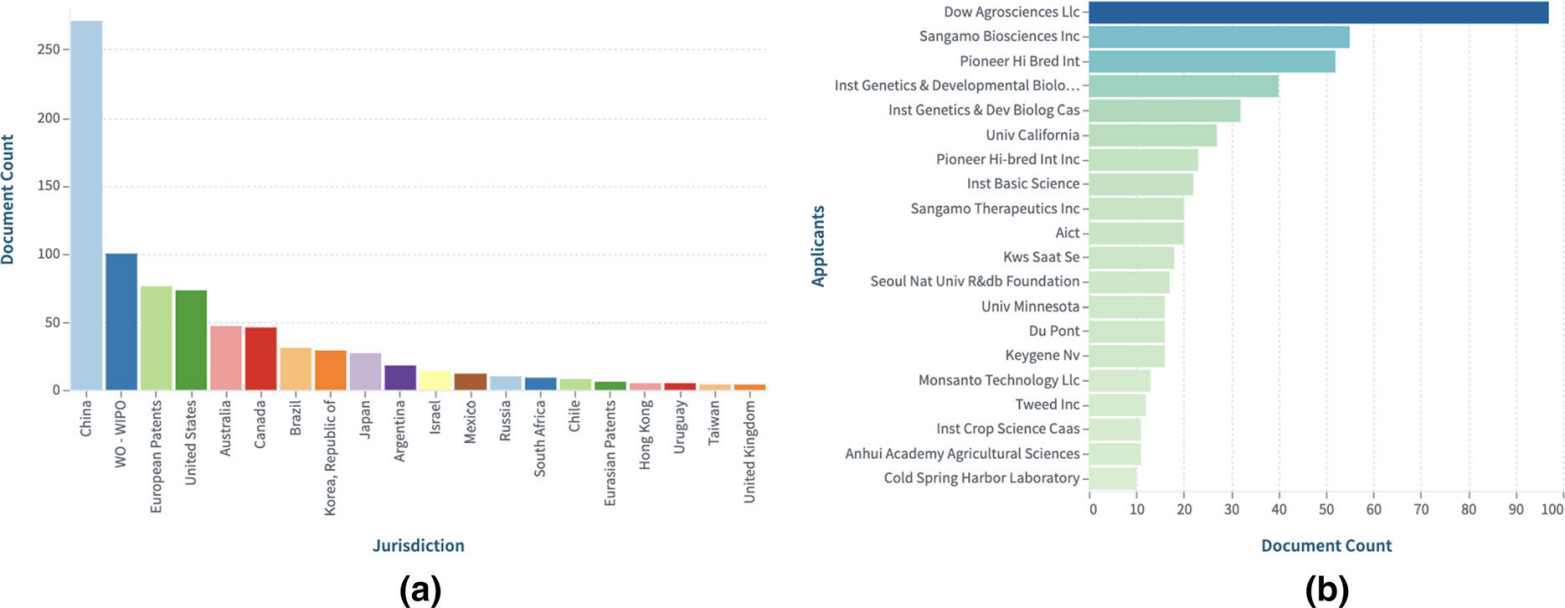
**a** Top jurisdiction based on expanded patent families, and **b** Top applicants based on expanded patent families. Explore further CRISPR:Plants-Expanded by simple patent family in the Lens.

### Trends in "Agriculture/Farm animals and Aquaculture" category (https://link.lens.org/5GV081SpSMi) See Fig. 14

**Fig. 14:**
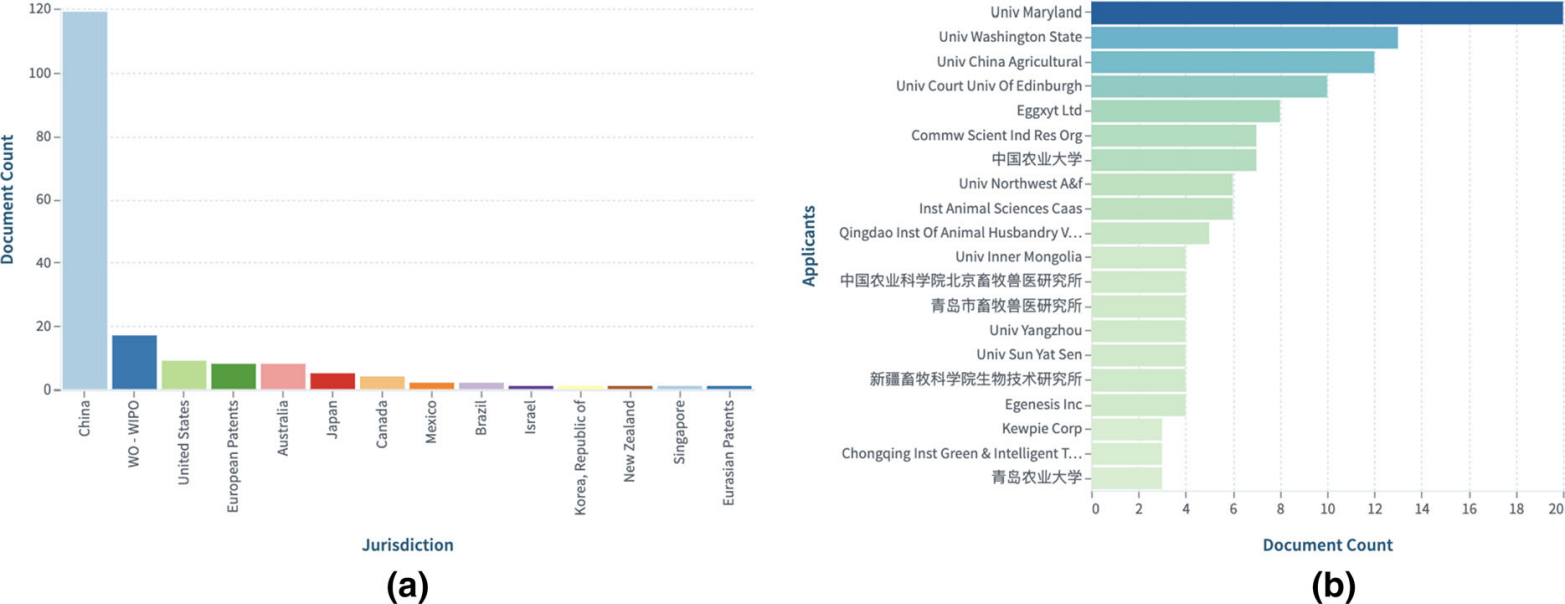
**a** Top jurisdictions based on expanded families, and **b **Top applicants based on expanded patent families. Explore further CRISPR:Farm animals and aquaculture-Expanded by simple patent family in the Lens.

## Data sources

- Scholarly work Collections
  1. CRISPR Cas9: Broad Items: 36,245, Created: Jun 19, 2020, Updated: Oct 9, 2020. Search string:
    title:CRISPR-Cas9 OR abstract:CRISPR-Cas9 OR keyword:CRISPR-Cas9 OR field_of_study:CRISPR-Cas9 OR title:cas9 OR abstract:cas9 OR keyword:cas9 OR field_of_study:cas9 OR (title:(CRISPR) OR abstract:(CRISPR) OR keyword:(CRISPR) OR field_of_study:(CRISPR)).
  2. CRISPR Cas9: Field of study CRISPR + Cas9 Items: 19,867, Created: Oct 8, 2020, Updated: Oct 8, 2020. CRISPR Cas9: Broad collection filtered by FIeld of Study: CRISPR and Cas9
  3. CRISPR Cas9: USA Items: 7,100, Created: Jun 19, 2020, Updated: Oct 9, 2020. CRISPR Cas9: Field of Study CRISPR + Cas9 filtered by USA
  4. CRISPR Cas9: China Items: 2,815, Created: Jun 19, 2020, Updated: Oct 9, 2020. CRISPR Cas9: Field of Study CRISPR + Cas9 filtered by China
  5. CRISPR Cas9: IP & Tech transfer Items: 47, Created: Sep 25, 2020, Updated: Oct 9, 2020. Search string: (title:("CRISPR Cas9") OR abstract:("CRISPR Cas9") OR fulltext:("CRISPR Cas9")) AND ((title:("Intellectual property") OR abstract: ("Intellectual property") OR fulltext:("Intellectual property")) OR (title:("IP landscape") OR abstract:("IP landscape") OR fulltext:("IP landscape")))
- Patent Collections
  1. CRISPR Cas9: Claims Items: 7,980, Created: Sep 23, 2020, Updated: Oct 8, 2020. Search string: claims:CRISPR OR claims:Cas9 OR claims:gRNAs OR claims:("RNAs guided") OR claims:("guided RNAs") OR claims:sgRNAs OR claims:("CRISPR cas9")
  2. CRISPR Cas9: only in claims Items: 2,908, Created: Oct 8, 2020, Updated: Oct 8, 2020. Search string: claims:(CRISPR Cas9)
  3. CRISPR Cas9: Claims & CPC codes Items: 3,627, Created: Jun 19, 2020, Updated: Oct 9, 2020. Search string: claims:(CRISPR cas9) OR classification_cpc:(C12N2310\/20*)
  4. CRISPR Cas9: Claims & CPC codes-Expanded Items: 8,844, Created: Jun 19, 2020, Updated: Oct 9, 2020. Search string: claims:(CRISPR cas9) OR classification_cpc:(C12N2310\/20*) expanded by simple patent family
- Collections reconstructed in the Lens from the supplementary information document in Martin-Laffon et al., 2019
  - Collections (one patent per family)
    1. CRISPR:Technical categories (families) Items: 2,070, Created: Mar 1, 2020, Updated: Oct 9, 2020. Based on Martin-Laffon, Kuntz & Ricroch (2019) patent list in the supplementary document of their publication. Out of 2072 patent families published, we recovered 2070 patent families when we grouped all the patents recovered from that list by the simple patent family facet
    2. CRISPR:Technical improvement (families) Items: 940, Created: Mar 1, 2020, Updated: Oct 9, 2020. This is based on the extracted list from the supplementary data by Martin-Laffon, Kuntz & Ricroch (2019) paper and grouped by patent family
    3. CRISPR: Medical (families) Items: 563, Created: Mar 2, 2020, Updated: Oct 9, 2020. The list created from Martin-Laffon, Kuntz & Ricroch (2019) supplementary data for this category was filtered by "group by simple patent family)
    4. CRISPR: Plants (families) Items: 261, Created: Mar 1, 2020, Updated: Oct 9, 2020. Based on extracted publication numbers from the supplementary list of Martin-Laffon et al., 2019 but grouped by patent family to get only one patent per family
    5. CRISPR: Industrial applications (families) Items: 168, Created: Mar 1, 2020, Updated: Oct 9, 2020. one patent per family and based on the published list in the supplementary data of Martin-Laffon et al. 2019
    6. CRISPR: Animals (families) Items: 86, Created: Mar 1, 2020, Updated: Oct 9, 2020. Publications keys from Martin-Laffon et al. 2019 supplementary list grouped by patent family
  - Expanded collections by patent family and based on the supplementary list of patents reports by Martin-Laffon et al. 2019 paper and then expanded by the simple patent family
    1. CRISPR: All technical categories-expanded Items: 8,867, Created: Oct 15, 2019, Updated: Oct 9, 2020
    2. CRISPR:Technical improvement-Expanded Items: 4,724, Created: Jun 19, 2020, Updated: Oct 9, 2020
    3. CRISPR: Health-Expanded Items: 2,282, Created: Jun 19, 2020, Updated: Oct 9, 2020
    4. CRISPR: Plants-Expanded Items: 806, Created: Oct 15, 2019, Updated: Oct 9, 2020
    5. CRISPR: Industrial Applications-Expanded Items: 644, Created: Jun 19, 2020, Updated: Oct 9, 2020
    6. CRISPR: Other in vitro-Expanded Items: 251, Created: Oct 15, 2019, Updated: Oct 9, 2020
    7. CRISPR: Farm Animals and aquaculture-Expanded Items: 179, Created: Oct 15, 2019, Updated: Oct 9, 2020

